# Supplementary material for: Dissecting fuel demand elasticities in Ghana: A quantile regression analysis using the Marshallian demand framework
Source: PLoS One. 2025 Oct 15;20(10):e0334540. doi: 10.1371/journal.pone.0334540 (PMC12527139; doi:10.1371/journal.pone.0334540)
Supplement: Attachment — Submitted filename: Response to Reviewers.docx [file pone.0334540.s002.docx]

**Dissecting fuel demand elasticities in Ghana: a quantile regression analysis using the Marshallian demand framework**

We sincerely thank the academic editor and reviewers for their constructive feedback and insightful comments on our manuscript titled. We have carefully considered each point raised and have made substantial revisions to address the concerns and suggestions provided. Below, we provide a point-by-point response to each comment where revisions have been implemented.

| **COMMENT** | **CORRECTION** |
| --- | --- |
| Title page must align PLOS ONE style | It has been corrected |
| Manuscript heading level and style | It has been corrected |
| Data availability | I have provided a link for data accessibility |
| Table 2 not referenced | All tables have been referred to in the manuscript |
| EEMD Justification: While EEMD is introduced as a noise-reduction technique, the manuscript could better explain why this method was chosen over alternatives (e.g., wavelet analysis) and how the selected IMFs (1, 5, residual) align with the study’s time horizons. | The most compelling advantage of the EEMD specifically addresses the mode mixing problem that plagued the original EMD. By adding white noise realizations and ensemble averaging, EEMD provides more meaningful IMFs that better separate different oscillatory components. Other methods like the wavelet decomposition, while avoiding mode mixing per se, can suffer from spectral leakage between adjacent frequency bands, particularly when analysing signals with closely spaced frequency components or rapid frequency variations. Also, EEMD maintains superior temporal localisation throughout the decomposition process, as each IMF preserves the original temporal resolution of the input signal, whiles other decomposition methods down sample at each decomposition level, potentially losing fine temporal details at higher frequency components.  Furthermore, IMF 1 represents short-term dynamics, capturing immediate consumer responses to price changes, seasonal variations, and monthly fluctuations where behavioural adjustments are limited by existing constraints. IMF 5 reflects medium-term structural adjustments spanning several time periods, encompassing business cycles, policy implementation effects, and moderate consumer adaptations such as vehicle choice changes or transportation mode shifts. The residual IMF also captures long-term trends and fundamental structural changes, including urbanization effects, economic development trajectories, and generational behavioural shifts that fundamentally alter fuel consumption patterns.  The selection of seven quantiles is grounded in statistical power considerations given the study's sample size of 275 monthly observations. QR requires adequate sample density within each conditional quantile to ensure reliable parameter estimation, and with 275 data points, seven quantiles provide approximately 39 observations per quantile segment, which meets the minimum threshold for stable coefficient estimation while avoiding small-sample bias that would emerge with more quantiles. Excessive quantile partitioning would lead to unstable estimates due to insufficient observations in the tail quantiles, while fewer than seven quantiles would compromise the study's ability to capture heterogeneous fuel demand responses across the conditional distribution, potentially masking critical variations in elasticity estimates. Therefore, the seven-quantile specification represents the optimal methodological approach that maximizes informational content while maintaining the statistical rigor necessary for reliable inference in QR analysis. |
| The exclusion of macroeconomic variables (e.g., exchange rates, global oil prices) and demographic factors (e.g., urbanization, vehicle ownership) is noted, but their potential impact on results could be discussed more critically. For instance, how might exchange rate fluctuations confound the observed price elasticities? | This study is limited by its exclusion of broader macroeconomic variables such as exchange rates and global oil prices, which may influence fuel demand. Exchange rate fluctuations present a particularly critical limitation, as Ghana's reliance on imported petroleum products even as an exporting country means currency depreciation affects domestic fuel prices independently of global oil movements, potentially confounding the estimated price elasticity coefficients by capturing currency-induced price variations rather than pure demand responsiveness. Global oil price dynamics may also bias elasticity estimates if international price volatility exhibits different temporal patterns than domestic market adjustments. The study also does not account for demographic or behavioural factors like vehicle ownership or urbanisation, which may mask heterogeneous demand responses across population segments, as urban consumers typically exhibit different elasticity patterns due to alternative transportation availability, while vehicle ownership directly influences fuel demand necessity. These exclusions may result in elasticity estimates that reflect aggregate behavioural responses rather than underlying structural demand relationships, potentially creating omitted variable bias and limiting policy relevance. Future research should integrate these variables and consider regional or cross-country comparisons to enhance generalisability and provide more robust estimates that account for the complex factors influencing energy consumption behaviours. |
| The non-stationarity of residuals in long-term analyses (e.g., QQR deviations) warrants deeper discussion. Are these deviations statistically significant, and do they affect policy conclusions? | The non-stationarity observed in the residuals for demand for petrol and price of diesel raises concerns about the reliability of estimated elasticity relationships, they suggest that the estimated fuel demand-price relationships may not be stable over the 275-month study period, potentially indicating structural breaks or time-varying elasticity parameters. This to some extent can significantly affect policy conclusions by making long-term forecasts unreliable and potentially rendering policy recommendations regarding fuel taxation or subsidy reforms ineffective. |
| The recommendations (e.g., differentiated subsidies, alternative energy investments) are sound but could be more specific to Ghana’s context.   - How might the government implement income-tiered pricing in practice? - What barriers exist to adopting electric vehicles/biofuels in Ghana, and how can they be addressed? - A brief comparison with successful policies in similar economies (e.g., Nigeria, Kenya) could strengthen the policy section. | The government could implement income-tiered fuel pricing through digital subsidy systems linked to national ID cards basically the Ghana card, where low-income households receive allocated subsidised fuel while higher earners pay market rates, or through progressive taxation where initial monthly allocations are subsidised with subsequent purchases taxed at increasing rates.  This can be addressed through public-private charging network partnerships, import duty exemptions, and grid improvements.  Ghana can emulate Kenya's successful implementation of a 10% ethanol blending mandate combined with comprehensive clean energy tax incentives by adopting a phased approach that integrates subsidy reforms with robust social safety nets. This strategy should incorporate regional biofuel cooperation frameworks that leverage West African agricultural resources and shared technological expertise while addressing Ghana's specific infrastructural limitations and economic constraints. |
| Figures/Tables: Some figures (e.g., 3D plots in Figure 2) are visually dense and could benefit from clearer labels or annotations to highlight key takeaways. | Corrected. |
| Terminology: Acronyms (e.g., IMFs, QQR) should be defined at first use, and technical terms (e.g., "inferior good") could be briefly explained for interdisciplinary readers. | The acronyms were defined at first use already.  Definition of terms were done in the methodology section. |
| Data Availability: The manuscript states data are "fully available without restriction," but no repository or DOI is provided. Clarifying where data can be accessed would enhance reproducibility. | A DOI have been provided in the manuscript for data accessibility. |
| References: Some citations lack titles or DOIs (e.g., [6], [22]), reducing traceability. Ensure all references follow journal guidelines. | Reference [6], does not have a DOI, it is a report and not an article.  Reference [20] DOI have been provided. |
